# Resistance after Chronic Application of the HDAC-Inhibitor Valproic Acid Is Associated with Elevated Akt Activation in Renal Cell Carcinoma In Vivo

**DOI:** 10.1371/journal.pone.0053100

**Published:** 2013-01-23

**Authors:** Eva Juengel, Jasmina Makarević, Igor Tsaur, Georg Bartsch, Karen Nelson, Axel Haferkamp, Roman A. Blaheta

**Affiliations:** 1 Department of Urology, Johann Wolfgang Goethe-University, Frankfurt am Main, Germany; 2 Department of Vascular and Endovascular Surgery, Johann Wolfgang Goethe-University, Frankfurt am Main, Germany; Johns Hopkins University, United States of America

## Abstract

Targeted drugs have significantly improved the therapeutic options for advanced renal cell carcinoma (RCC). However, resistance often develops, negating the benefit of these agents. In the present study, the molecular mechanisms of acquired resistance towards the histone deacetylase (HDAC) inhibitor valproic acid (VPA) in a RCC in vivo model were investigated. NMRI:nu/nu mice were transplanted with Caki-1 RCC cells and then treated with VPA (200 mg/kg/day). Controls remained untreated. Based on tumor growth dynamics, the mice were divided into “responders” and “non-responders” to VPA. Histone H3 and H4 acetylation and expression of cell signaling and cell cycle regulating proteins in the RCC mouse tumors were evaluated by Western blotting. Tumor growth of VPA responders was significantly diminished, whereas that of VPA non-responders even exceeded control values. Cdk1, 2 and 4 proteins were strongly enhanced in the non-responders. Importantly, Akt expression and activity were massively up-regulated in the tumors of the VPA non-responders. Chronic application (12 weeks) of VPA to Caki-1 cells in vitro evoked a distinct elevation of Akt activity and cancer cells no longer responded with cell growth reduction, compared to the short 2 week treatment. We assume that chronic use of an HDAC-inhibitor is associated with (re)-activation of Akt, which may be involved in resistance development. Consequently, combined blockade of both HDAC and Akt may delay or prevent drug resistance in RCC.

## Introduction

Renal cell carcinoma (RCC) is the most common renal tumor with an incidence of 11.8 per 100,000 in industrialized nations [1]. Although the majority of patients with clinically localized tumors can effectively be cured, those with metastatic RCC have a bleak prognosis. During the last decade, intensive efforts have been undertaken to detect tumor specific molecules, with the hope that pharmacologic blockade of these proteins may counteract neoplastic progression. Epigenetic changes have been shown to be induced by abnormal histone deacetylase (HDAC) activity and to correlate with tumor development and progression. Immunohistologic assessment of 44 RCC cases have provided evidence that decreased histone acetylation is a common alteration in the malignant phenotype of this tumor entity [2]. Tissue microarray analysis carried out on 193 patients with RCC revealed an inverse correlation between histone acetylation and pT-stage, distant metastasis, Fuhrman grading and RCC progression [3]. Based on clinical data, it has been suggested that increasing the amount of acetylated histones by lowering HDAC might be a therapeutic option for RCC. In fact, in vitro and in vivo experiments point to distinct growth and invasion blocking properties of HDAC-inhibitors in RCC models [4]–[6]. Unfortunately, the therapeutic benefit demonstrated in pre-clinical studies has not satisfactorily been affirmed in clinical trials [7], [8] and may be due to the patients having acquired resistance during long-term drug treatment.

Therefore, tumor growth, histone acetylation status and expression of cell signaling and cell cycle regulating proteins were compared in RCC cell bearing mice, some of which respond and some of which do not respond to chronic treatment with the HDAC-inhibitor valproic acid (VPA). Evidence is presented that the tumors in non-responders are characterized by a massive up-regulation of Akt expression and activity. Additional in vitro experiments demonstrated that Akt re-activation occurs during long-term VPA treatment.

## Materials and Methods

### Ethics statement

All animal experiments were performed according to the German Animal Protection Law and by approval of the local responsible authorities (Approval Number: A0452/08; Ethics Committee of the Landesamt für Gesundheit und Soziales, Berlin, Germany).

### Kidney carcinoma Caki-1 cells

RCC Caki-1 cells were purchased from LGC Promochem (Wesel, Germany). The cells were grown and subcultured in RPMI 1640 medium (Seromed, Berlin, Germany) supplemented with 10% FCS, 100 IU/ml penicillin and 100 µg/ml streptomycin at 37°C in a humidified, 5% CO_2_ incubator.

### Tumor growth in vivo under chronic VPA application

10^7^ Caki-1 cells (100 µl volume) were subcutaneously injected into male NMRI:nu/nu mice (EPO GmbH, Berlin, Germany). VPA treatment was initiated when tumors had grown to a palpable size (5–6 mm diameter). VPA (G. L. Pharma GmbH, Lannach, Austria) was dissolved in 100% peanut oil and injected once daily i.p. at a dose of 200 mg/kg/day (n = 6) for 63 days. The control group received solvent (n = 6). Tumor size was measured with calipers. Tumor volume, relative tumor volume (relative to the first treatment day) and treated/control (T/C) values were calculated. Body weight and mortality were recorded continuously to determine tolerability. Animals were sacrificed by CO_2_ ventilation at the humane endpoint, i.e. as soon as the first animal displayed a 2 cm3 sized tumor (occurred 63 days after tumor cell injection) and tissue specimens from the nude mice xenografts were collected and frozen. The expression of cell cycle regulating and target proteins was evaluated by Western blot analysis.

### VPA application to Caki-1 cells in vitro

Cultured Caki-1 cells were exposed to 1 mM VPA (diluted in cell culture medium) twice a week. The treatment procedure lasted for 2 versus 12 weeks, after which the cells were subjected to the MTT assay for 24 h, 48 h and 72 h, or to the Western blot assay to detect Akt and pAkt. Control cell cultures were not exposed to VPA. For the MTT assay, 1×104 cells/ml were seeded onto 96-well tissue culture plates (100 µl/well) in cell culture medium including VPA (controls received cell culture medium alone). After 24, 48 and 72 h, MTT (0.5 mg/ml) was added for an additional 4 h. Thereafter, cells were lysed in a buffer containing 10% SDS in 0.01 M HCl. The plates were incubated overnight at 37°C, 5% CO2. Absorbance at 570 nm was determined for each well using a microplate ELISA reader. Each experiment was done in triplicate. After subtracting background absorbance, results were expressed as mean cell number. Cell number at 24 h was set to 100%.

The 2 week application protocol was carried out to resemble early effects of drug treatment, i.e. tumor cells still sensitive to VPA. The 12 week application protocol reflects chronic drug treatment over a longer period of time, when tumor cells begin to acquire drug non-responsiveness. Cells treated for 2 weeks are designated “Short-term”, cells treated for 12 weeks are designated “Long-term” in the figures.

### Blocking studies

The function blocking monoclonal antibody Akt inhibitor VIII (Akti-1/2; Chemdea, Ridgewood, NJ, USA), was used to block Akt1 and Akt2 activity in one set of Caki-1 cells (incubated for 1 h with 1 µM). These cells were designated Akt^low^ cells. Another cell set was incubated with cell culture medium alone and served as the non-blocked cells, designated Akt^high^ cells. Both Akt^high^ and Akt^low^ cells were then treated with 1 mM VPA and immediately subjected to the MTT growth assay. Akt^high^ and Akt^low^ control cells were not exposed to VPA. In an additional experiment, the Caki-1 cells were cultured in serum-free medium for 24 h. The serum-free medium was then replaced by medium containing serum, and tumor cells were activated by 100 ng/ml insulin-like growth factor I (IGF) for another 24 h. Subsequently, Akt^high^ and Akt^low^ cells were exposed to VPA as described above.

### Caki-1 cell growth in vitro (MTT growth assay)

Cell growth of Akt^high^ versus Akt^low^ cells was assessed using the 3-(4,5-dimethylthiazol-2-yl)-2,5-diphenyltetrazolium bromide (MTT) dye reduction assay (Roche Diagnostics, Penzberg, Germany). The Akt^high^ and Akt^low^ Caki-1 cells (50 µl, 1×10^5^ cells/ml) were seeded onto 96-well tissue culture plates. After 24 h and 120 h, 10 µl MTT (0.5 mg/ml) was added for an additional 4 h. Thereafter, cells were lysed in a buffer containing 10% SDS in 0.01 M HCl. The plates were allowed to stand overnight at 37°C, 5% CO_2_. Absorbance at 550 nm was determined for each well using a microplate ELISA reader. Each experiment was done in triplicate. After subtracting background absorbance, results were expressed as mean cell number. The difference between the 24 h and the 120 h cell number was calculated to obtain the 96 h cell increase. 96 h control values (Akt^high^ and Akt^low^ cells not treated with VPA) were set at 100%.

### Western blot analysis

To explore cell cycle regulating proteins in the mouse tumors, cell lysates were applied to a 7–15% (depending on protein size) polyacrylamide gel and electrophoresed (90 min, 100 V). The protein was then transferred to nitrocellulose membranes (1 h, 100 V). After blocking with non-fat dry milk for 1 h, the membranes were incubated overnight with monoclonal antibodies directed against cell cycle proteins: cdk1 (IgG1, clone 1), cdk2 (IgG2a, clone 55), cdk4 (IgG1, clone 97), cyclin A (IgG1, clone 25), cyclin B (IgG1, clone 18), cyclin D1 (IgG1, clone G124–326), cyclin E (IgG1, clone HE12), Rb (IgG2a, clone 2), p21 (IgG1, clone 2G12), p27 (IgG1, clone 57), p53 (IgG2b, clone DO-7; all: mouse, BD Biosciences, Heidelberg, Germany) and p73 (IgG1, clone ER-15, Dianova GmbH, Hambourg, Germany).

To explore target specificity of VPA, histone acetylation and HDAC inhibition were evaluated. The following monoclonal antibodies were used: Histone H3 (rabbit, clone 3H1), acetyl-Histone H3 (Lys9, rabbit, polyclonal), Histone H4 (mouse, IgG*K*, clone L64C1), acetyl-Histone H4 (Lys8, rabbit, polyclonal, all: Cell Signaling Technology, Danvers, MA, US), Histone Deacetylase 3 (rabbit, polyclonal) and Histone Deacetylase 4 (rabbit, polyclonal, both: Biomol International, Lörrach, Germany).

Relevant signaling proteins involved in proliferation of Caki-1 cells in vitro were also examined. The following antibodies were used: Akt (IgG1, clone 55), phosphoAkt (pAkt, IgG1, clone 104A282, both: mouse, BD Biosciences, Heidelberg, Germany), p70S6 Kinase (rabbit, IgG, clone 49D7), phospho p70S6 Kinase (pp70S6K, rabbit, IgG, clone 108D2), PTEN (rabbit, polyclonal), phospho PTEN (pPTEN, rabbit, IgG, clone 44A7, all: Cell Signaling Technology, Danvers, MA, USA).

HRP-conjugated goat-anti-mouse IgG and HRP-conjugated goat-anti-rabbit IgG (both: Upstate Biotechnology, Lake Placid, NY, USA) served as the secondary antibody. The membranes were briefly incubated with ECL detection reagent (ECL™, Amersham/GE Healthcare, München, Germany) to visualize the proteins and then analyzed by the Fusion FX7 system (Peqlab, Erlangen, Germany). ß-actin (Sigma, Taufenkirchen, Germany) served as the internal control.

### Statistics

All experiments were performed 3–6 times. Statistical significance was determined with the Wilcoxon–Mann-Whitney-U-test. Differences were considered statistically significant at a p-value less than 0.05.

## Results

### Chronic application of VPA induces resistance in vivo

**Figure 1:**
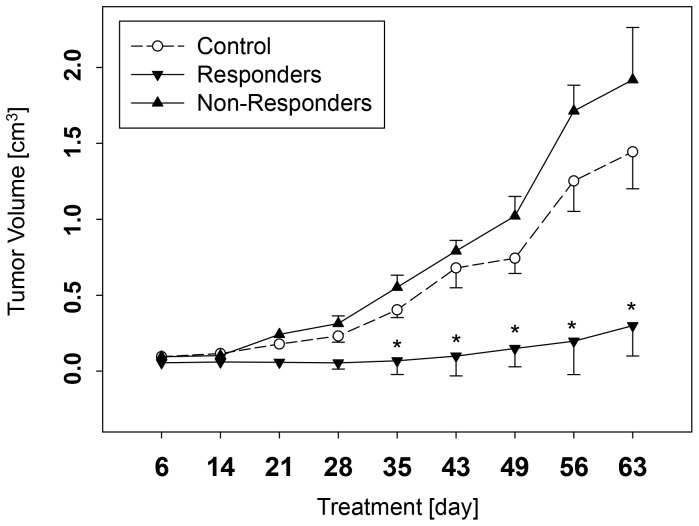
Effect of VPA on tumor volume in Caki-1 xenografts in mice. Mice in the treatment arm received 200 mg VPA/kg once daily. Control mice received solvent (n = 6). *indicates significant difference to the control mice.

All animals survived. Long-term application of VPA resulted in significant inhibition of tumor cell growth in 50% of the mice. The remaining 50% did not respond to VPA at all. The tumor mass in the non-responsive cohort was even slightly enhanced, compared to untreated controls (fig. 1). Mice with distinct tumor reduction, compared to controls, were designated ‘VPA responders’, whereas mice with no tumor reduction under VPA were defined as ‘VPA non-responders’.

### Cell cycle protein expression in responders versus non-responders

**Figure 2:**
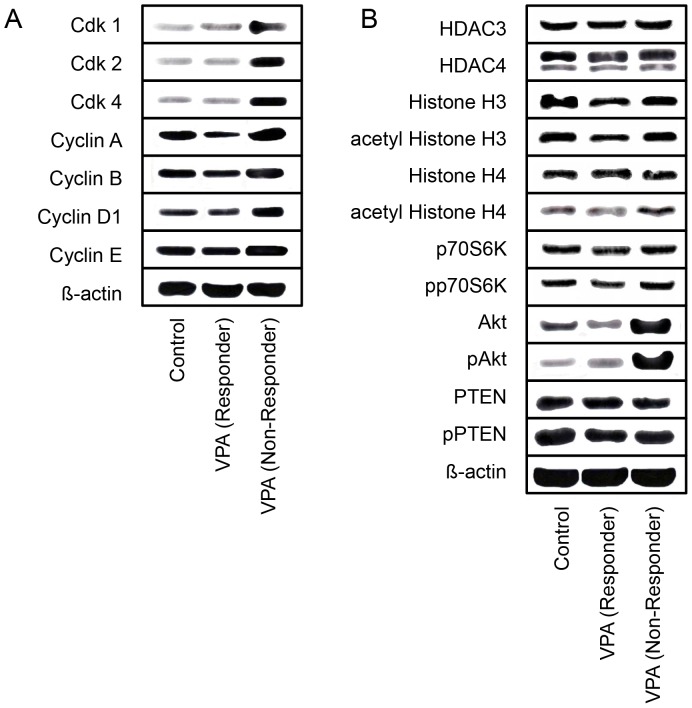
Western Blot analysis of cell cycle regulating (fig. 2A) and cell signaling proteins (fig. 2B) in tumor tissue from drug-sensitive (responders) versus drug-resistant mice (non-responders). Control tissue specimens were taken from untreated animals. Cell lysates (50 µg) were subjected to SDS-PAGE and blotted on the membrane incubated with the corresponding monoclonal antibodies. β-actin served as the internal control. The figure shows one representative from three separate experiments.

To gain insight into the molecular mechanism of resistance development, cell cycle regulating proteins were analyzed. Expression of cdk1, cdk2 and cdk4 in the VPA responders was similar to the controls. However, cyclin A was profoundly reduced, and cyclin B, cyclin D1 and cyclin E were slightly diminished in the responders group. In contrast, cdk1, cdk2 and cdk4, together with cyclin A and D1, were strongly up-regulated in the VPA non-responders. Additionally, cyclin B and E were moderately elevated, compared to the control animals (fig. 2A).

### Intracellular signaling in responders versus non-responders

HDAC and histone analysis were done after 10 weeks of chronic VPA exposure. No differences in HDAC expression (both HDAC3 and HDAC4) were seen in treated versus non-treated animals. A moderate reduction of total and acetylated histone H3 was evident in responders, whereas the H3 and aH3 level were not altered in the non-responders, compared to the control (fig. 2B). The tumor suppressor and Akt-inhibitor PTEN, additionally evaluated, was down-regulated in VPA non-responders. Most importantly, a massive up-regulation of Akt (both total and activated) was evoked in the non-responders. In responders there was a small increase of pAkt, whereas total Akt was strongly diminished.

### Relevance of Akt in VPA resistance development

**Figure 3:**
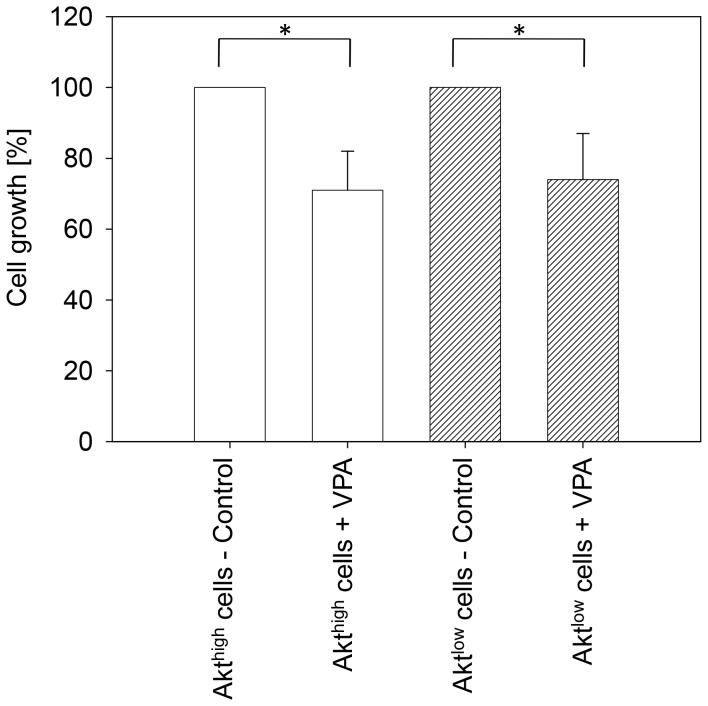
VPA acts on Caki-1 growth, independent of the initial Akt expression level. Akt^high^ and Akt^low^ expressing Caki-1 cells, which were generated by a function blocking anti-Akt monoclonal antibody, were treated with 1 mM VPA (controls remained untreated) and subjected to the MTT cell growth assay (figure 3A). One representative from 6 experiments. *indicates significant difference to controls.

Since Akt was profoundly elevated in tumor cells resistant to VPA treatment, the question arose as to whether differing initial Akt expression levels might be responsible for VPA non-responsiveness. In a first series of experiments, Akt was blocked by a function blocking monoclonal antibody in cultured Caki-1 cells (Akt^low^) or left unblocked (Akt^high^) and the efficacy of VPA in suppressing cell growth was evaluated. VPA exposure caused cell growth to be diminished by 29±4% in Akt^high^ cells and by 27±6% in Akt^low^ cells (fig. 3), showing that the anti-growth potential of VPA is not dependent on the initial Akt expression level. The same lack of difference in cell growth in Akt^high^ and Akt^low^ RCC cells was found when, pre-activated with IGF, they were subjected to the cell growth assay (data not shown).

**Figure 4:**
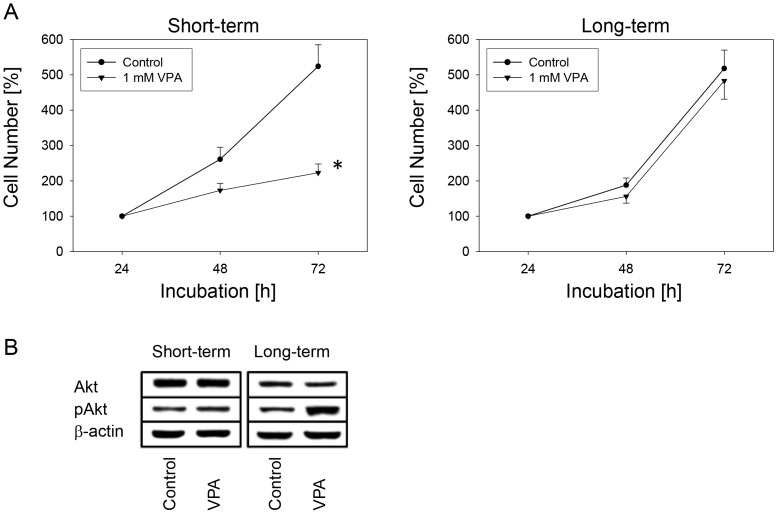
Long-term exposure of VPA causes drug resistance and Akt up-regulation. Fig. 4A: Caki-1 cells were treated for 2 weeks (short-term) or 12 weeks (long-term) with 1 mM VPA, and cell growth was analysed by the MTT assay. Controls remained untreated. The figure shows one representative from six separate experiments. *indicates significant difference to controls. Fig. 4B: To evaluate Akt expression and activity, Caki-1 cells were treated short-term or long-term with 1 mM VPA. Controls remained untreated. Cell lysates were subjected to SDS-PAGE and blotted on the membrane incubated with the respective monoclonal antibodies. β-actin served as the internal control. One representative from three separate experiments.

In a second set of experiments, short-term treatment of cultured Caki-1 cells with VPA for two weeks resulted in a significant reduction of cell growth, whereas long-term treatment over 12 weeks was associated with drug insensitivity (fig. 4A) and a profound increase in Akt expression (fig. 4B).

## Discussion

Although HDAC-inhibition has led to a distinct reduction of cancer growth and invasion in preclinical studies, patient trials have provided mixed results. Epigenetic therapy, consisting of adding hydralazine and VPA to one of the current standard combination chemotherapies for cervical cancer, has demonstrated a significant advantage in progression-free survival [9]. The combination of vorinostat and tamoxifen has been encouraging in reversing hormone resistance in patients with metastatic breast cancer [10]. No objective response, but prolonged stable disease, was observed in a study including patients with several advanced solid tumor malignancies treated with entinostat in combination with 13-cis retinoic acid [11]. However, additive use of panobinostat in patients with solid tumors did not consistently inhibit HDAC activity [12].

The reason for the clinical insufficiency of HDAC-inhibitors is not clear to date. Based on an in vivo RCC model, evidence is presented here showing that chronic VPA application causes resistance. The in vivo data have been corroborated by in vitro studies revealing resistance acquisition with long-term VPA exposure. Therefore, it seems plausible that failure of an HDAC-inhibitor based regimen might be due to resistance development.

Molecular analysis has revealed a massive up-regulation of cdk and cyclin type proteins in drug resistant RCC. Cdk-cyclin complexes operate as the major cell signaling components in all stages of the cell cycle. Nevertheless, only limited data are available dealing with the role of these molecules in RCC. Immunohistochemical investigation of RCC tissue samples demonstrated cyclin D1 and D3 expression to be closely associated with tumor size, stage and grade [13], [14]. The corresponding partner, cdk4, was particularly linked to von Hippel-Lindau negative RCC [15]. A uni- and multivariate statistical analysis indicated the significant role of cyclin B in RCC development and pathogenesis [16]. There is also evidence that high cyclin A expression is an unfavorable prognostic factor in patients with RCC [13].

Resistance development caused by an HDAC-inhibitor based regimen is, at least partially, characterized by a distinct accumulation of cdk/cyclin proteins, which may re-activate the cell cycle machinery. RCC cells chronically treated with VPA for 12 weeks in vitro have been shown to increase cyclin A and cyclin D3 expression and to simultaneously regain the capacity to grow [17]. However, quantitative modification of cdk proteins was not observed in this model. Possibly, the in vitro conditions differ from the in vivo one presented here and identical results cannot be expected. Studies investigating the relevance of cdk-cyclin complexes in drug resistant RCC cells are necessary.

The VPA-induced resistance could be due to increased levels of HDAC accompanied by reduced histone acetylation. However, neither HDAC3/HDAC4, nor H3/H4 acetylation was altered in the drug resistant mice, compared to the untreated control. This is important, since the HDAC system would be the specific target of an HDAC-inhibitor. Obviously, a feedback mechanism in the course of resistance development has not been established, leading to an up-regulation of HDAC and down-regulation of histone acetylation. Recently, resistance to the HDAC-inhibitor SAHA has been reported not to be accompanied by elevated expression of HDAC1 and HDAC3 in human colorectal adenocarcinoma cells [18]. However, this does not mean that HDAC is irrelevant during the process of resistance induction. The following aspect must also be considered: VPA enhances histone H3 and H4 acetylation in RCC cells at a very early time point. This effect is lost following long-term exposure. The H3 and H4 acetylation levels are then similar to the expression level of untreated control cells [17]. Hypothetically, resistance to VPA might be defined by the failure to up-regulate histone acetylation (rather than by the feedback mechanism to diminish histone acetylation).

The most prominent effect of VPA was a massive amplification of Akt expression and activity in the non-responders as demonstrated by western blotting, which did not occur in the untreated mice. Akt plays a central role in the control of cell growth, survival and angiogenesis, whereby aberrant activation and dysfunction becomes evident in progressive RCC [19], [20]. Due to this relationship, blocking Akt and Akt downstream molecules by mammalian target of rapamycin (mTOR) inhibitors has been considered an effective strategy in fighting this disease. Indeed, mTOR-suppression has produced robust clinical effects in RCC, particularly in the early treatment phase. However, compensatory Akt (re)activation seems to be a critical event under long-term application, which may limit the antitumor effect of mTOR-inhibitors [21]. The data presented here demonstrates that up-regulation of Akt is not a resistance phenomenon exclusively restricted to the use of mTOR-inhibitors, but may also occur in the presence of HDAC-inhibitors. This property could label Akt as a ubiquitous prognostic and therapeutic parameter for patients subjected to targeted drugs. Recently, resistance of colon cancer cells to the HDAC-inhibitor butyrate has been demonstrated to be coupled to high Akt levels [22].

The molecular mechanism responsible for VPA non-responsiveness is not yet clear. Based on in vivo results, two hypotheses are conceivable: 1) RCC cells are initially equipped with a huge mass of highly activated Akt, which counteracts the antigrowth potential of VPA exerted by HDAC-inhibition. Since altering the Akt level in RCC cells in vitro did not influence the efficacy of VPA to diminish growth, this hypothesis seems unlikely. 2) Chronic VPA application induces Akt elevation in RCC cells over time, finally leading to drug non-responsiveness. The in vitro studies presented here, conducted with therapeutically relevant VPA concentrations, provide evidence that Akt rises with long-term VPA treatment of RCC cells, which negatively correlates with the capacity of VPA to stop cancer growth. In another experimental setting, prolonged exposure of gastric cancer cells to increasing concentrations of butyrate resulted in the acquisition of resistance, which was accompanied by Akt up-regulation [22]. Furthermore, the sensitivity of lung adenocarcinoma cells to the HDAC-inhibitor FK228 inversely depends on the Akt signaling pathway [23]. Therefore, the RCC cells may establish undesired feedback loops in the presence of VPA. Akt may serve as the dominant counter regulator, finally enabling the cancer cells to restart their growth program.

This opens the question of whether combined inhibition of HDAC and Akt may prevent VPA driven resistance induction. Chronic application of either VPA or the mTOR-inhibitor everolimus has caused drug non-responsiveness in RCC cells, which however, could be prevented when both agents were used in combination [17]. A novel strategy has been provided by Qian and coworkers to overcome the dynamic and adaptive natures of tumor cells. They constructed a dual-acting compound by incorporating HDAC inhibitory functionality into an Akt inhibitor pharmacophore. Greater growth inhibition and proapoptotic activity than single-target Akt- or HDAC- inhibitors in both cultured and implanted cancer cells was shown [24]. Disrupting cancer networks via simultaneous interference with the Akt pathway and epigenetic effects on HDAC may, therefore, offer improved therapeutic benefits in RCC.

This study reveals that chronic HDAC-inhibition resulted in drug non-responsiveness of RCC cells. Resistance development was accompanied by elevated Akt expression and activity, indicating a cross-link between HDAC and the Akt-pathway. Ongoing in vivo studies are necessary to verify whether a combined HDAC- and Akt-inhibitor based regimen might circumvent or delay the onset of acquired resistance.
